# Supplementary material for: Important role of endogenous microbial symbionts of fish gills in the challenging but highly biodiverse Amazonian blackwaters
Source: Nat Commun. 2023 Jul 6;14:3903. doi: 10.1038/s41467-023-39461-x (PMC10326040; doi:10.1038/s41467-023-39461-x)
Supplement: Supplementary file 1 — Supplementary Information [file 41467_2023_39461_MOESM1_ESM.pdf]

Supplementary material

Important role of endogenous microbial symbionts of fish gills in the challenging but highly biodiverse Amazonian blackwaters

Sylvain, François-Étienne[12]; Leroux, Nicolas[1]; Normandeau, Eric[1]; Custodio, Jaqueline[3]; Mercier, Pierre-Luc[1]; Bouslama, Sidki[1]; Holland, Aleicia[4]; Barroso, Danilo[3†]; Val, Adalberto Luis[3]; Derome, Nicolas[1]

1. Institut de Biologie Intégrative et des Systèmes, Université Laval, 1030 avenue de la Médecine, Québec (QC), G1V 0A6, Canada
2. Fisheries and Oceans, Gulf Fisheries Center, 343 University Ave, Moncton, NB E1C 5K4
3. Instituto Nacional de Pesquisas da Amazônia (INPA), Laboratório de Ecofisiologia e Evolução Molecular, Manaus, AM, 69067-375, Brasil
4. La Trobe University, School of Agriculture, Biomedicine and Environment, Department of Environment and Genetics, Centre for Freshwater Ecosystems, Albury/Wodonga Campus, Vic, Australia

† in memorian

Supplementary tables

Suppl. Table 1: Site identification, water color, geographical coordinates, ecosystem type and sampling time for each sampling site.

Site characteristics						
**Site #**	**Site name**	**Water color**	**GPS S**	**GPS W**	**Ecosystem**	**Sampling time**
1	Rio Negro - Barcelos	Black	0°50'50.8"S	62°57'40.3"W	River	11/2018
2	Rio Negro - Santo Alberto	Black	1°23'29.8"S	61°59'35.3"W	River	10/2019
3	Rio Negro - Anavilhanas	Black	2°41'46.1"S	60°46'33.3"W	River	10/2018
4	Lago do cemeterio	Black	3°02'16.6"S	60°32'42.7"W	Lake	10/2019
5	Lago Téfé	Black	3°27'55.2"S	64°53'13.2"W	Lake	11/2019
6	Rio Branco	White	1°19'05.7"S	61°52'34.7"W	River	10/2019
7	Lago Janauari	White	3°12'03.4"S	60°03'10.1"W	Lake	10/2018
8	Lago Catalão	White	3°09'56.4"S	59°54'38.4"W	Lake	10/2018
9	Lago Janauaca	White	3°23'37.5"S	60°19'52.6"W	Lake	11/2018
10	Rio Manacapuru	White	3°16'16.9"S	60°42'03.2"W	River	11/2018
11	Lago Téfé-Solimões	White	3°21'07.4"S	64°40'21.4"W	Lake	11/2019
12	Lago des pirates	White	3°15'19.2"S	64°41'44.3"W	Lake	11/2019
13	Balbina Reservoir	Clear	1°50'55.9"S	59°34'59.5"W	Reservoir	10/2018
14	Rio Tapajós	Clear	2°18'57.8"S	55°00'45.0"W	River	10/2019
15	Rio Curua-Una	Clear	2°48'19.1"S	54°17'52.2"W	River	11/2018

Suppl. Table 2: Sequencing output of Amazonian fish host gill transcriptomes (NovaSeq).

**Site**	**Number of reads per species per site**			
	**Flag cichlid**	**Black piranha**	**Sardine**	**Peacock bass**
Site #1	1.48E+08	3.18E+08	2.26E+08	2.45E+08
Site #2	4.28E+08	2.81E+08	6.07E+08	1.86E+08
Site #3	3.22E+08	3.75E+08	4.20E+08	3.66E+08
Site #4	2.19E+08	3.23E+08	2.85E+08	2.17E+07
Site #5	2.99E+08	4.35E+08	3.19E+08	1.78E+08
Site #6	3.11E+08	2.81E+08	3.46E+08	7.22E+07
Site #7	3.21E+08	2.00E+08	3.27E+08	1.73E+08
Site #8	2.97E+08	1.97E+08	3.06E+08	5.71E+07
Site #9	1.95E+08	1.85E+08	3.83E+08	3.58E+07
Site #10	2.40E+08	-	-	1.30E+08
Site #11	2.39E+08	1.97E+08	2.05E+08	1.40E+08
Site #12	2.84E+08	3.66E+08	3.44E+08	1.52E+08
Site #13	1.36E+08	1.06E+08	2.68E+08	9.33E+07
Site #14	6.12E+07	1.31E+08	2.12E+08	-
Site #15	1.18E+08	2.33E+08	1.80E+08	-
Total	3.62E+09	3.63E+09	4.43E+09	1.85E+09
Global total	1.35E+10			

Suppl. Table 3: Sequencing output of zebrafish host gill transcriptomes (NovaSeq).

Water color	Treatment	Reads
Black water	BWIS	7.73E+07
	BWIS	8.25E+07
	BWN	1.75E+08
	BWS	1.34E+08
White water	WWIS	6.52E+07
	WWIS	1.81E+08
	WWN	2.28E+08
	WWS	1.07E+08
Total		1.05E+09

Suppl. Table 4: Number of specimens of each host species collected at each sampling site[1].

Site #	*M. festivus*	*T. albus*	*S. rhombeus*	*Cichla spp.*
1	20	20	20	18
2	20	11	20	17
3	20	20	20	5
4	20	0	0	11
5	20	18	17	4
6	20	20	20	0
7	20	16	15	18
8	20	20	20	20
9	20	20	20	20
10	20	20	20	17
11	20	13	12	0
12	20	15	15	0
13	20	9	7	19
14	20	14	20	13
15	20	20	20	20
Total	300	236	246	182

***1:** "*M. festivus*" stands for *Mesonauta festivus*, "*T. albus*" stands for *Triportheus albus* and "*S. rhombeus*" stands for *Serrasalmus rhombeus*.

Supp. Table 5: Measure of DOC quantity and FDOM optical characteristics[1].

Site #	DOC and FDOM characteristics						
	DOC conc.	SAC340	SUVA254	Abs 254/365	% humic FDOM	% fulvic FDOM	% protein FDOM
1	10.9	39.5	4.5	3.8	56.7	29.5	13.8
2	11.7	33.5	3.7	3.6	60.3	32.6	7.1
3	11.4	30.5	3.6	3.8	47.2	30.3	22.5
4	9.8	18.9	2.4	3.8	52.3	41.0	6.7
5	7.1	29.1	3.4	4.0	54.2	37.3	8.5
6	6.0	19.1	2.2	4.3	50.8	39.7	9.5
7	7.1	19.1	1.4	2.2	34.7	36.2	29.1
8	9.1	11.7	2.1	6.4	37.5	45.6	16.8
9	5.7	20.0	2.6	4.2	50.6	40.4	9.0
10	8.0	22.1	3.0	4.6	46.2	41.8	12.0
11	5.7	20.1	2.6	3.8	49.0	39.3	11.7
12	6.5	14.2	2.2	4.7	43.9	45.3	10.8
13	4.9	6.1	1.2	7.1	30.6	42.3	27.1
14	2.7	8.7	1.9	5.0	44.8	38.3	16.9
15	4.6	11.7	1.9	5.3	35.1	45.0	20.0

***1:** "DOC" stands for dissolved organic carbon, "FDOM" stands for fluorescent dissolved organic matter, "DOC conc." means DOC concentration in mg L^{-1}. SAC340 and SUVA254 are the specific absorbance coefficients index of relative DOM aromaticity (higher the values more aromatic is the DOM). Abs254/365 is the index of molecular weight: The lower the value the higher molecular weight is the DOM is.

Suppl. Table 6: Concentrations of free ions and nutrients.

Site #	Water color	Ions: mg L^{-1}					Nutrients: umol L^{-1}		
		Na^{+}	Mg^{+2}	K^{+}	Ca^{+2}	Cl^{-}	Nitrite	Nitrate	Silicate
1	Black	0.46	0.12	0.42	0.04	0.11	0.11	3.20	64.41
2	Black	0.25	0.09	0.33	0.49	1.16	0.10	2.87	92.32
3	Black	1.80	0.26	0.65	0.08	0.32	0.09	4.36	72.55
4	Black	0.23	0.05	0.14	0.37	0.64	0.01	0.47	76.82
5	Black	0.87	0.19	0.56	0.82	0.53	0.08	4.09	217.19
6	White	1.15	0.43	0.70	0.93	1.10	0.04	8.23	180.48
7	White	1.99	0.20	0.79	0.06	1.47	0.19	1.31	98.31
8	White	4.56	3.76	1.71	0.83	1.75	0.09	0.56	242.31
9	White	3.32	1.00	1.07	0.44	2.17	0.13	20.45	156.51
10	White	4.91	0.14	1.45	0.05	1.43	0.12	1.53	126.01
11	White	1.95	0.21	0.28	1.11	1.29	0.03	6.47	326.53
12	White	5.35	1.76	1.28	1.17	3.26	0.61	11.96	222.31
13	Clear	0.80	0.14	0.67	0.03	0.78	0.05	1.55	85.93
14	Clear	0.43	0.47	0.57	0.68	0.39	0.09	1.91	179.36
15	Clear	1.52	0.26	0.67	0.04	1.22	0.06	2.55	171.96

Suppl. Table 7: Primary productivity characterization and measure of physicochemical parameters.[1]

Site #	Water color	Primary productivity: ug L^{-1}			Physicochemical parameters			
		Chl a	Phaeopig.	Chla/DOC	Temp. °C	Cond. uS	pH	% O_2
1	Black	0.35	2.43	0.03	31.60	13.10	3.71	92.12
2	Black	0.73	0.33	0.06	30.60	10.60	4.16	58.00
3	Black	0.05	0.38	0.00	30.70	13.20	4.24	53.20
4	Black	1.35	1.44	0.14	32.40	7.20	3.83	76.90
5	Black	1.82	1.73	0.26	30.00	10.60	4.98	61.50
6	White	6.21	2.89	1.03	31.00	22.00	6.25	88.70
7	White	4.62	17.31	0.65	32.90	22.40	4.38	60.00
8	White	7.14	6.60	0.79	32.90	174.80	5.70	44.00
9	White	1.35	1.88	0.24	29.30	88.00	6.75	82.60
10	White	2.78	10.54	0.35	32.60	24.30	5.31	72.80
11	White	4.41	3.20	0.77	30.30	19.70	6.05	68.60
12	White	9.05	4.69	1.40	31.90	127.60	7.15	31.90
13	Clear	0.83	0.78	0.17	33.20	16.80	5.05	103.20
14	Clear	2.15	1.03	0.81	30.00	14.10	6.36	80.20
15	Clear	1.25	2.38	0.28	31.20	19.00	6.00	79.10

***1:** "Chl a" means the concentration of chlorophyll a; "Phaeopig." means the concentration of phaeopigments; "Chla/DOC" is the ratio of chlorophyll a concentration divided by DOC concentration; "Temp. °C" means the temperature in ° Celsius; "Cond. uS" means the conductivity in microsiemens; "% O_2" means the percentage of saturation of dissolved oxygen.

Suppl. Table 8: Concentration of dissolved metals in ug/L.

Site #	Water color	Metals (ug/l)											
		Al	V	Cr	Mn	Fe	Co	Ni	Cu	Zn	As	Cd	Pb
1	Black	137.75	0.38	0.30	7.38	166.63	0.13	1.93	10.36	33.48	0.16	0.09	1.43
2	Black	150.00	0.10	0.05	5.90	160.00	0.10	0.15	0.30	11.00	0.05	0.02	0.27
3	Black	36.33	0.34	0.37	9.24	142.38	0.28	3.23	9.25	72.92	0.48	0.21	1.11
4	Black	87.00	0.30	0.05	4.60	100.00	0.10	0.33	1.90	9.00	0.08	0.13	0.30
5	Black	62.00	0.10	0.33	13.00	220.00	0.10	0.52	0.60	4.40	0.19	0.02	0.12
6	White	38.00	0.20	0.05	0.51	230.00	0.10	0.14	0.80	2.60	0.07	0.02	0.26
7	White	65.50	0.78	0.40	9.85	269.28	0.10	0.85	16.19	44.15	0.47	0.06	0.67
8	White	1.81	0.17	0.10	0.61	5.84	0.10	0.48	2.20	171.78	0.99	0.02	0.03
9	White	28.02	1.45	0.09	11.25	166.97	0.10	0.58	2.73	1.83	0.70	0.03	0.25
10	White	13.47	0.85	0.21	4.64	97.85	0.10	1.12	2.11	25.85	0.38	0.08	0.16
11	White	49.00	0.30	0.11	0.68	250.00	0.10	0.41	0.50	2.70	0.27	0.02	0.21
12	White	27.00	0.20	0.06	4.60	82.00	0.10	0.60	1.70	8.10	1.30	0.02	0.11
13	Clear	10.29	0.05	0.05	0.23	16.85	0.10	0.13	0.56	4.49	0.14	0.02	0.05
14	Clear	5.00	0.10	0.05	0.05	7.00	0.10	0.10	0.50	3.70	0.07	0.02	0.03
15	Clear	18.49	0.17	0.58	12.31	52.88	0.11	1.18	2.12	23.94	0.64	0.07	0.25

Suppl. Table 9: Comparison of water parameters measured in the original sample and after transport to the laboratory (prior to the axenic zebrafish experiment).[1]

		Blackwater Santo Alberto		Whitewater Lago des pirates	
Parameter	Measure	Original sample	After transport	Original sample	After transport
[Al]	µg/L	150.00	113.00	27.00	11.00
[As]	µg/L	<0.30	<0.30	1.30	0.90
[Cd]	µg/L	<0.10	<0.10	<0.10	<0.10
[Ca^{+2}]	µg/L	490.00	127.00	1170.00	1530.00
[Cr]	µg/L	<0.50	<0.50	<0.50	<0.50
[Co]	µg/L	<0.50	<0.50	<0.50	<0.50
[Cu]	µg/L	0.30	12.60	1.70	9.40
[Fe]	µg/L	160.00	175.00	82.00	38.00
[Mg^{+2}]	µg/L	90.00	55.00	1176.00	2320.00
[Mn]	µg/L	5.90	4.00	4.60	1.00
[Ni]	µg/L	<1.00	<1.00	<1.00	<1.00
[Pb]	µg/L	0.27	<0.10	0.11	<0.10
[K^{+}]	µg/L	<500.00	<500.00	1280.00	1300.00
[Na^{+}]	µg/L	250.00	430.00	5350.00	4490.00
[V]	µg/L	<0.50	<0.50	<0.50	<0.50
[Zn]	µg/L	11.00	4.00	8.10	<3.00
[DOC]	mg/L	11.67	10.40	6.47	5.84
Conductivity	µS	11.00	22.00	128.00	128.00
pH	-	4.16	4.05	7.15	7.00

***1:** "DOC" stands for dissolved organic carbon.

Suppl. Table 10: Proteins known to be involved in ionoregulatory processes in fish facing acidic and ion-poor environments.[1]

Protein	Strategy	Reference
Na^{+}/H^{+} antiporters (NHE)	Strategy #2	Morris *et al.* 2021
V-type proton ATPases	Strategy #2	Morris *et al.* 2021
Sodium channels	Strategy #2	Morris *et al.* 2021
Rh glycoproteins (Rhag, Rhbg, Rhcg)	Strategy #2	Morris *et al.* 2021
Carbonic anhydrases	Strategy #2	Morris *et al.* 2021
Na^{+}/K^{+} ATPases	Strategy #2	Morris *et al.* 2021
Sodium/Potassium/Calcium exchangers	Strategy #2	Morris *et al.* 2021
Electrogenic Na^{+}/HCO3^{-} cotransporters	Strategy #2	Morris *et al.* 2021
Cl^{-}/HCO_3^{-} exchangers	Strategy #2	Guh *et al.* 2015
Anion exchangers (e.g. Cl^{-} exchanger)	Strategy #2	Guh *et al.* 2015
Chloride channels	Strategy #2	Guh *et al.* 2015
Calcium channels	Strategy #2	Guh *et al.* 2015
Potassium channels	Strategy #2	Guh *et al.* 2015
Na^{+} Cl^{-} cotransporters	Strategy #2	Guh *et al.* 2015
Na^{+} Ca_2^{+} exchangers	Strategy #2	Guh *et al.* 2015
Ca_2^{+} ATPases	Strategy #2	Guh *et al.* 2015
Solute carriers (SLC)	Strategy #2	Guh *et al.* 2015
Occludins	Strategy #1	Araujo *et al.* 2017
Claudins	Strategy #1	Araujo *et al.* 2017
Actinin	Strategy #1	Araujo *et al.* 2017
Integrins	Strategy #1	Araujo *et al.* 2017
Desmoplakins	Strategy #1	Araujo *et al.* 2017
Gap junction proteins	Strategy #1	Araujo *et al.* 2017
Glucocorticoid receptors	Strategy #1	Araujo *et al.* 2017
Prolactin receptors	Strategy #1	Araujo *et al.* 2017
Mineralocortocoid receptors	Strategy #1	Chasiotis *et al.* 2012
Zonula occludens (plaque proteins)	Strategy #1	Chasiotis *et al.* 2012
Protein kinase C	Strategy #1	Ulluwishewa *et al.* 2011
Mitogen-activated protein kinase	Strategy #1	Ulluwishewa *et al.* 2011
Myosin light chain kinase	Strategy #1	Ulluwishewa *et al.* 2011
Rho family of small GTPases (RhoA, Rac, Cdc42, etc)	Strategy #1	Ulluwishewa *et al.* 2011
Rho kinases	Strategy #1	Ulluwishewa *et al.* 2011
Tricellulins	Strategy #1	Ulluwishewa *et al.* 2011
JAM proteins (JAM-A, coxsackie/adenovirus receptor)	Strategy #1	Ulluwishewa *et al.* 2011
Toll-like receptors	Strategy #1	Ulluwishewa *et al.* 2011
Cadherins	Strategy #1	Ghosh *et al.* 2021

Catenins	Strategy #1	Ghosh *et al.* 2021
Desmogleins	Strategy #1	Ghosh *et al.* 2021
Desmocollins	Strategy #1	Ghosh *et al.* 2021
Connexins	Strategy #1	Ghosh *et al.* 2021

***1:** Strategy #1 refers to the regulation of ion efflux and strategy #2 refers to the regulation of active ion uptake.

Supplementary figures

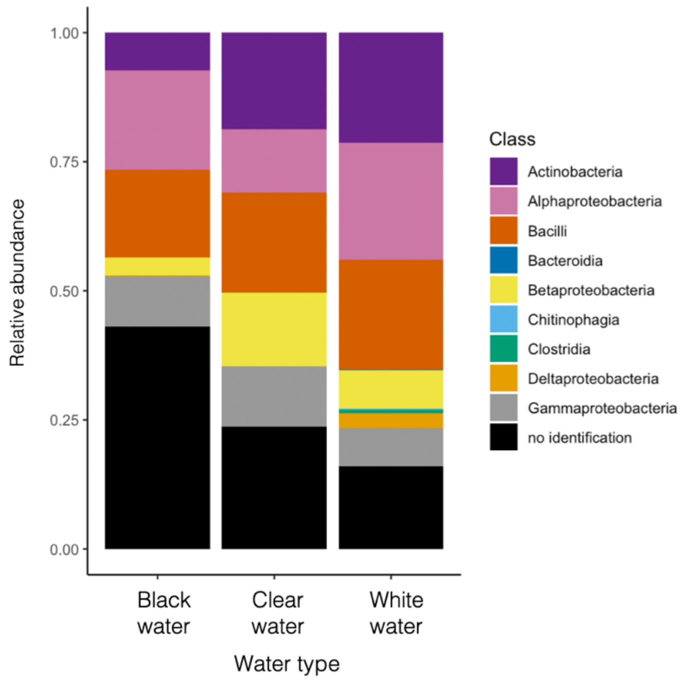

Suppl. Fig. 1: Stacked barplot of the relative abundance of the bacterial classes detected in the bacterioplankton samples collected at the 15 Amazonian field sampling sites (16S rRNA gene dataset). Source data are provided as a Source Data file.

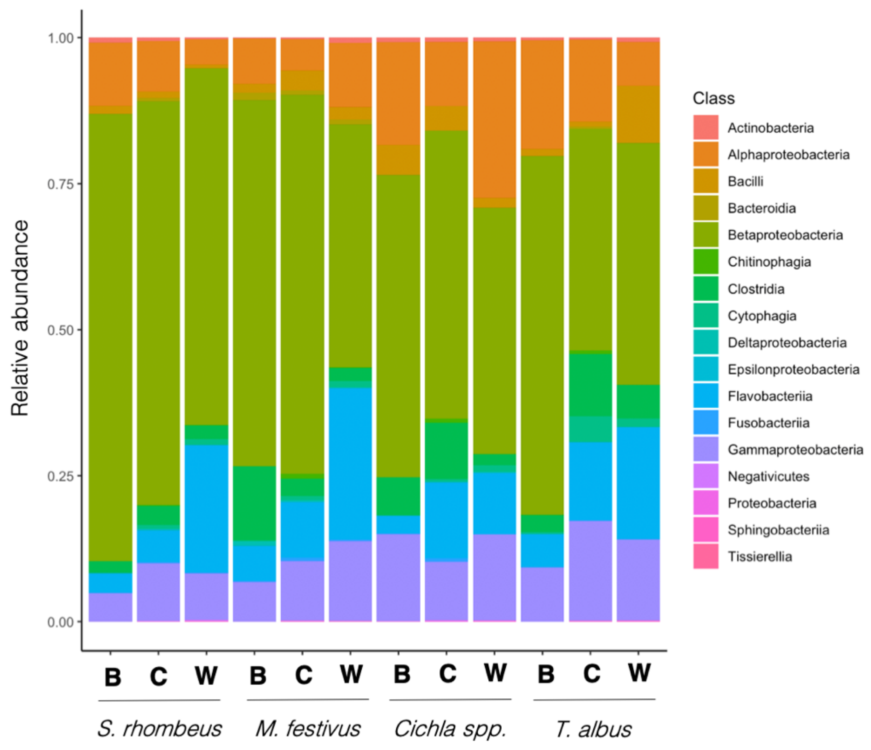

Suppl. Fig. 2: Stacked barplot of the relative abundance of the bacterial classes detected in the Amazonian fish gills microbiomes (16S rRNA gene dataset). "*M. festivus*" stands for *Mesonauta festivus*, "*T. albus*" stands for *Triportheus albus* and "*S. rhombeus*" stands for *Serrasalmus rhombeus*. The letters in bold indicate water type: "B" for blackwater, "C" for clearwater and "W" for whitewater. Source data are provided as a Source Data file.

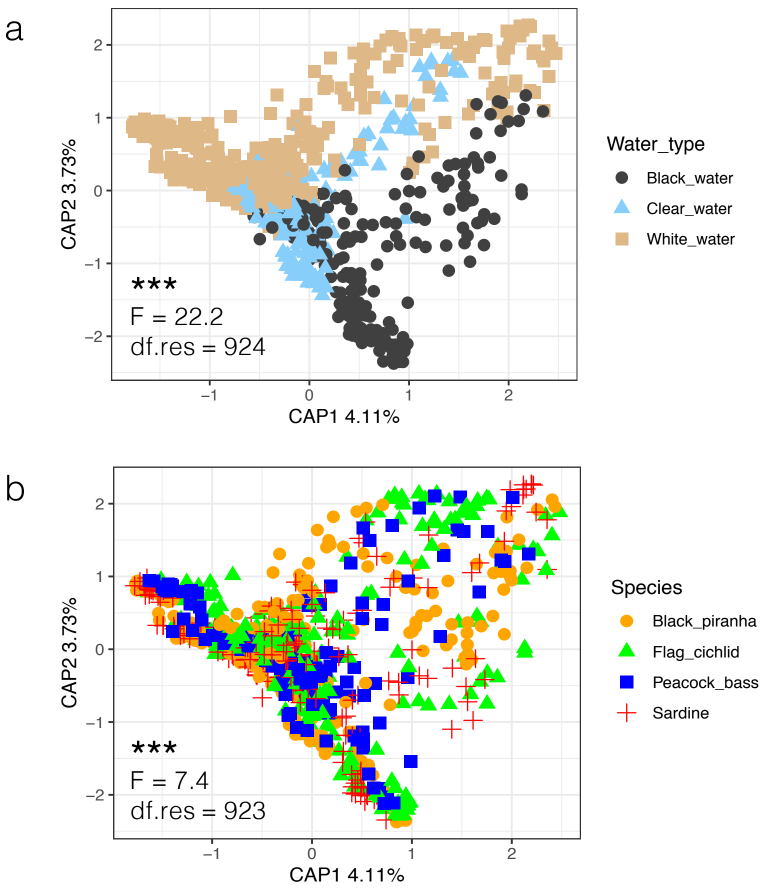

Suppl. Fig. 3: Bray-Curtis distance-based redundancy analyses with all samples together (all species and water types). Samples are colored according to their water type in (a) and to their host species in (b). Permutational analysis of variance (PERMANOVA, 999 permutations, two-sided test) results for each factor are at the bottom left of the corresponding plots. *** means p-value < 0.001. df.res means the residual degrees of freedom. Overall, the results show that both water type and host species are significantly associated to microbiome composition (PERMANOVA p-values < 0.001 for both factors), however the water type factor is approximately 3x stronger than the host species factor (F water type = 22.2 versus F host species = 7.4). Source data are provided as a Source Data file.

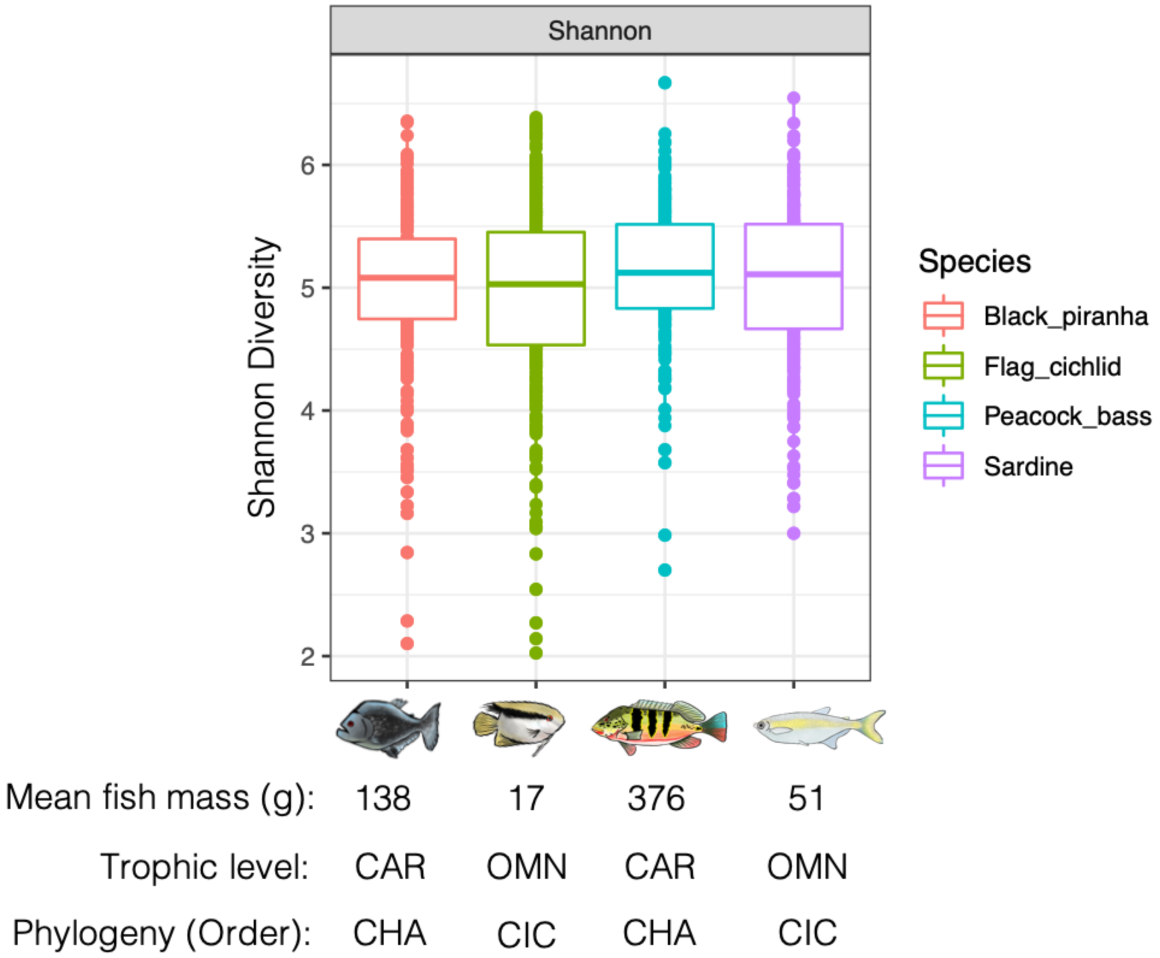

Suppl. Fig. 4: Shannon diversity measure of gill microbiome samples from the four host species collected. “CAR” stands for carnivorous; “OMN” for omnivorous; “CHA” for Characiforms; “CIC” for Cichliformes. Gill microbiome Shannon diversity was not significantly different between host species, trophic levels or host phylogeny (Order) and was not significantly correlated to mean fish mass (g). Biologically independent samples of black piranha (N=231), flag cichlid (N=296), peacock bass (N=172), and freshwater sardine (N=228) microbiotas were examined. The center of the boxplots correspond to the median. The lower and upper bounds of boxes represent the first and third quartiles (the 25th and 75th percentiles). The lower whiskers extend from the lower bounds of boxes to the lowest values within 1.5 * the inter-quartile range (distance between the first and third quartiles). The upper whiskers extend from the upper bounds of boxes to the highest values that is within 1.5 * the inter-quartile range. Data beyond the end of the whiskers are outliers and plotted as points. Source data are provided as a Source Data file.

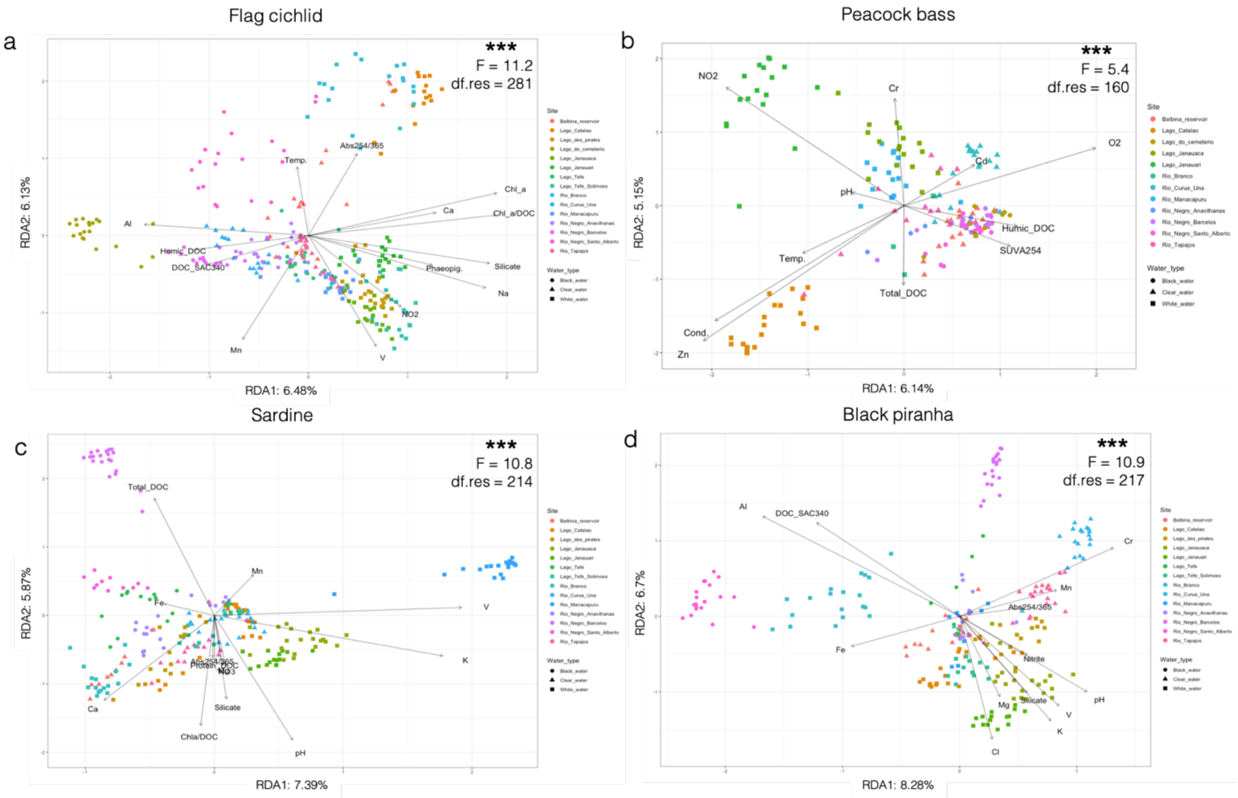

Suppl. Fig. 5: Bray-Curtis distance-based redundancy analyses (dbRDA) on the gill bacterial (16S rRNA) community samples. The analysis was conducted on samples from (a) flag cichlid, (b) peacock bass, (c) sardine, and (d) black piranha. Each data point in the dbRDAs represents a microbiome sample. The samples are colored according to their sampling site of origin and their shape corresponds to the water type of the site sampled. The environmental parameters represented in the dbRDAs were selected by *ordistep* and had a VIF < 10. PERMANOVA results (999 permutations, two-sided test) exploring the differences in community composition between sampling sites are at the upper-right corner of each plot. "***" stands for PERMANOVA p-value < 0.001. df.res stands for residual degrees of freedom. Overall, the results show that the sampling site factor is significantly associated to microbiome composition (PERMANOVA p-values < 0.001) for each host species. Among other factors, differences in the physicochemical parameters between sites of the same water type might partly explain the effect of the sampling site factor (e.g. conductivity, pH , and concentrations of Al, Fe and Zn could explain differences between different clusters of whitewater sites, Suppl. Tables 7 and 8). To prevent confounding the water type and sampling site effects, fish were sampled from a minimum of three sampling sites per water type. Source data are provided as a Source Data file.

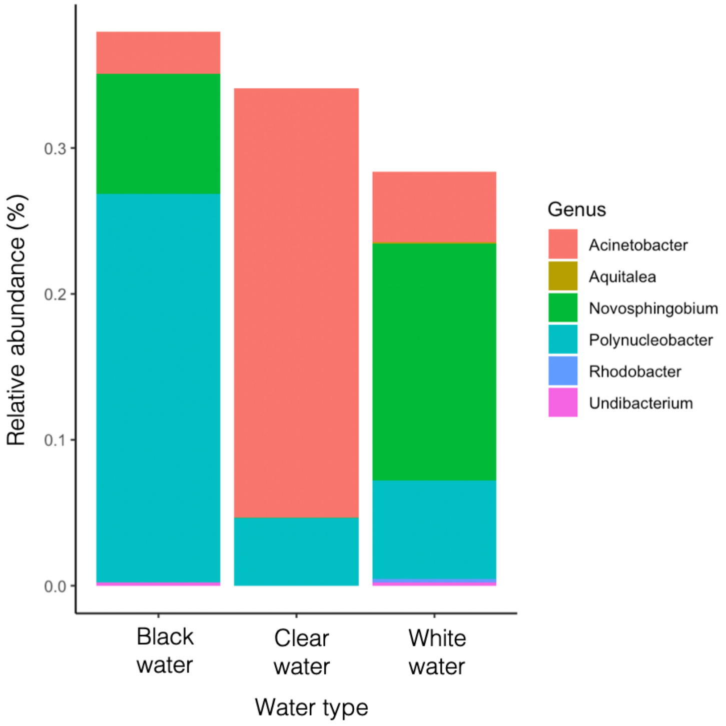

Suppl. Fig. 6: Stacked barplot showing the relative abundance of the fish gill bacterial biomarkers in the free-living bacterioplanktonic communities collected at the 15 Amazonian field sampling sites (16S rRNA gene dataset). The bacterial biomarkers not shown were not detected in the bacterioplankton. Source data are provided as a Source Data file.

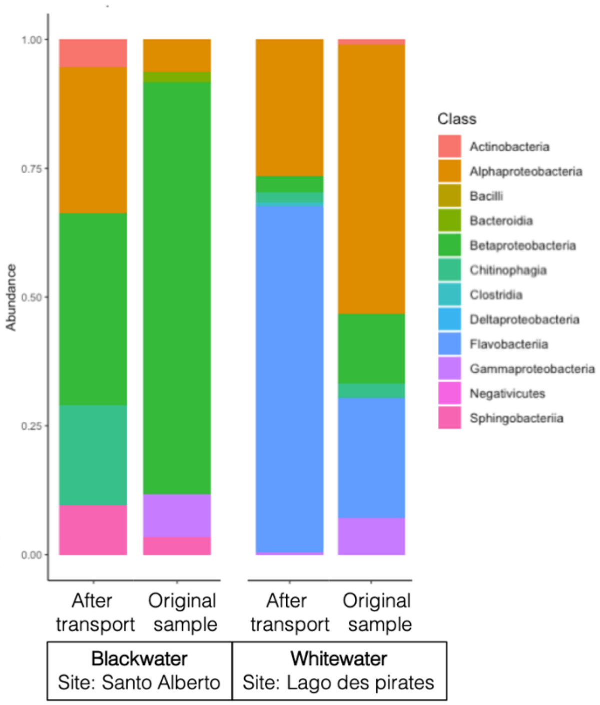

Suppl. Fig. 7: Stacked barplot of the relative abundance of the bacterial classes detected in water samples used for the axenic zebrafish experiment (16S rRNA dataset). The “Original sample” was taken directly on site, and the “After transport” sample was taken after transportation of the water from the field to the laboratory, right before the start of the axenic zebrafish experiment. In the blackwater from the Santo Alberto site, the sample taken after transportation shows a decrease in Betaproteobacteria and Gammaproteobacteria, and an increase in Actinobacteria, Alphaproteobacteria and Chitinophagia. In the whitewater from Lago des pirates, the sample taken after transportation shows an increase in Flavobacteriia, and a decrease in Proteobacteria (Alphaproteobacteria, Betaproteobacteria and Gammaproteobacteria). Source data are provided as a Source Data file.

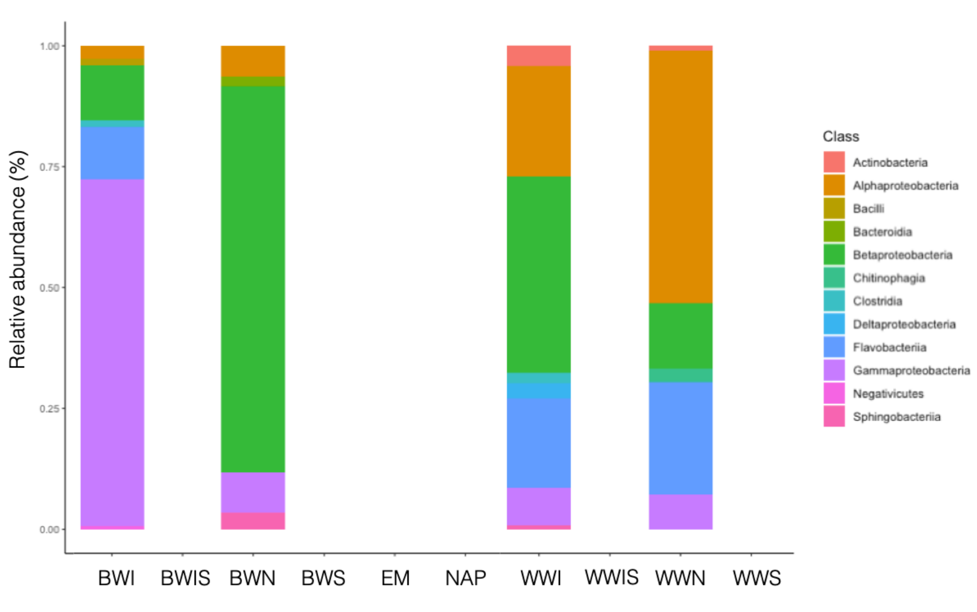

Suppl. Fig. 8: Stacked barplot of the relative abundance of the bacterial classes detected in the **water samples** collected during the axenic zebrafish experiment (16S rRNA dataset from RNA extracts). ”BWI” stands for inverted non-sterile blackwater, “BWIS” for inverted sterile blackwater, “BWN” for non-sterile blackwater, “BWS” for sterile blackwater, “EM” for embryo medium (the medium in which zebrafish larvae were kept for the first 96h), “NAP” for nucleic acid preservation buffer (to control potential cross-contamination from the buffer), “WWI” for inverted non-sterile whitewater, “WWIS” for inverted sterile whitewater, “WWN” for non-sterile whitewater, and “WWS” stands for sterile whitewater. Overall, this plot shows that after filtration of potential contaminant sequences using the *decontam* R package (Davis *et al.,* 2018), and after removal of sequences appearing <2 times in at least one of the experimental groups, we do not detect any ASV in sterile and in control groups. Source data are provided as a Source Data file.

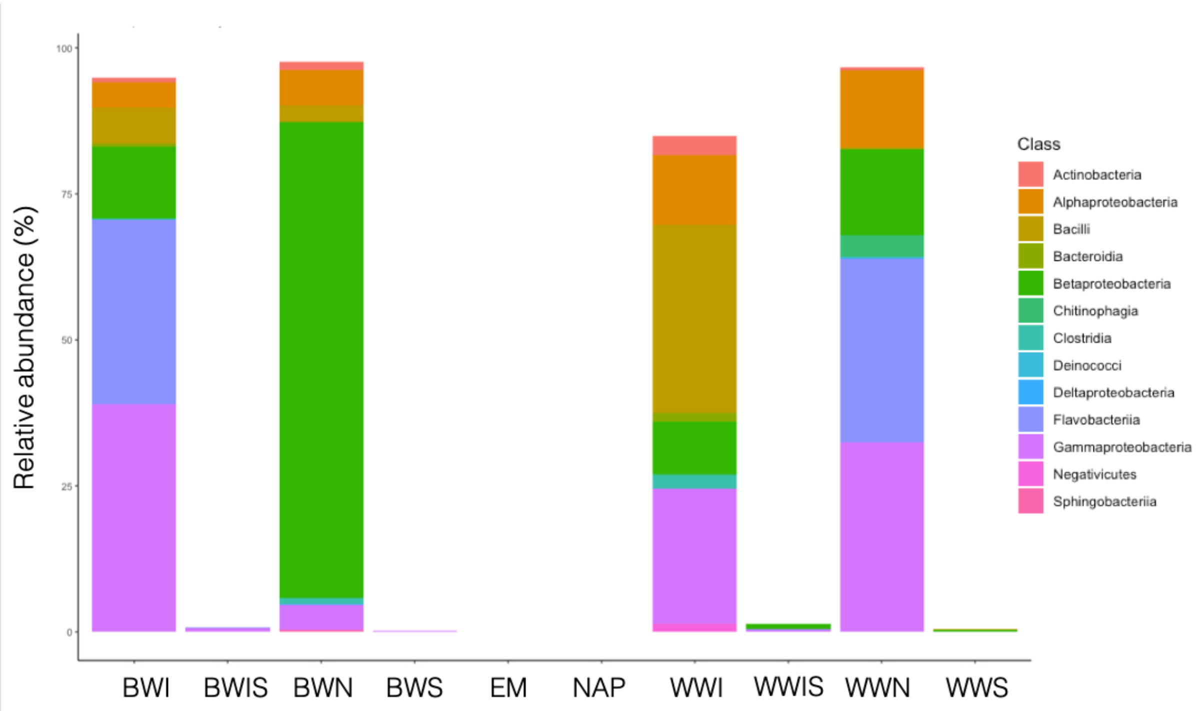

Suppl. Fig. 9: Stacked barplot of the relative abundance of the bacterial classes detected in the **zebrafish larvae samples** collected during the axenic zebrafish experiment (all groups in this plot except "EM" and "NAP" are fish samples) (16S rRNA dataset from RNA extracts). "BWI" stands for inverted non-sterile blackwater zebrafish, "BWIS" for inverted sterile blackwater zebrafish, "BWN" for non-sterile blackwater zebrafish, "BWS" for sterile blackwater zebrafish, "EM" for embryo medium (the medium in which zebrafish larvae were kept for the first 96h, shown here as control), "NAP" for nucleic acid preservation buffer (to control potential cross-contamination from the buffer, shown here as control), "WWI" for inverted non-sterile whitewater zebrafish, "WWIS" for inverted sterile whitewater zebrafish, "WWN" for non-sterile whitewater zebrafish, and "WWS" stands for sterile whitewater zebrafish. Overall, this plot shows that after filtration of potential contaminant sequences using the *decontam* R package (Davis *et al*., 2018), and after removal of sequences appearing <2 times in at least one of the experimental groups, we detect a very low number of ASVs in sterile and in control groups. Source data are provided as a Source Data file.

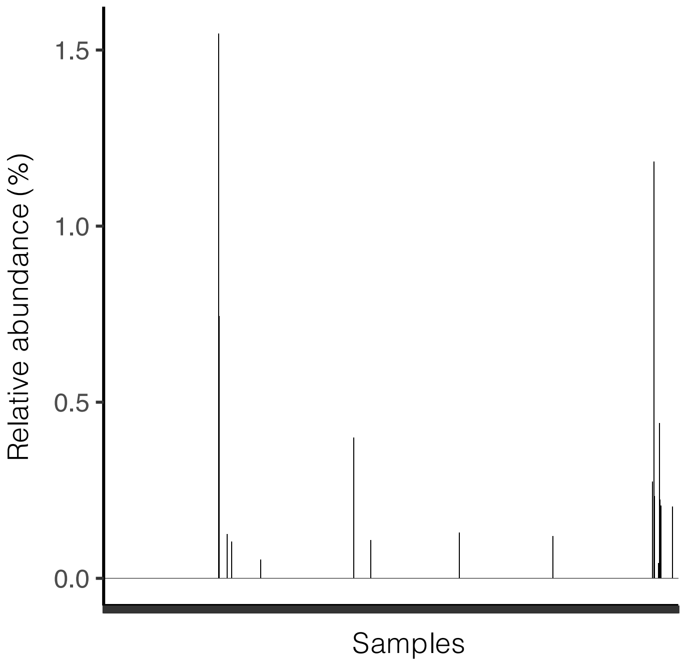

Suppl. Fig. 10: Barplot showing the relative abundance (in %) of the ASVs identified as potential contaminants (N = 61 ASVs) by the R package *decontam* (Davis *et al.* 2018). Negative controls and sterile samples were excluded from this analysis. The ASVs identified as contaminants had an average relative abundance of 0.007% and reached a maximum of 1.54% per sample. Source data are provided as a Source Data file.

References

1. Araújo, J. D. A., Ghelfi, A., & Val, A. L. *Triportheus albus* Cope, 1872 in the blackwater, clearwater, and whitewater of the Amazon: A case of phenotypic plasticity? *Front Genet* **8,** 114 (2016).

2. Chasiotis, H., Kolosov, D., Bui, P., & Kelly, S. P. Tight junctions, tight junction proteins and paracellular permeability across the gill epithelium of fishes: a review. *Resp physiol & neurobiol* **184(3),** 269–281 (2012).

3. Davis, N.M., *et al.* Simple statistical identification and removal of contaminant sequences in marker-gene and metagenomics data. *Microbiome* **6,** 226 (2018).

4. Ghosh, S., Whitley, C. S., Haribabu, B., & Jala, V. R. Regulation of intestinal barrier function by microbial metabolites. *Cell and Mol Gastroenterol and Hepatol*, **11(5)**, 1463–1482 (2021).

5. Guh, Y. J., Lin, C. H., & Hwang, P. P. Osmoregulation in zebrafish: Ion transport mechanisms and functional regulation. *EXCLI journal* **14,** 627–659 (2015).

6. Morris, C., Val, A. L., Brauner, C. J., & Wood, C. M. The physiology of fish in acidic waters rich in dissolved organic carbon, with specific reference to the Amazon basin: Ionoregulation, acid–base regulation, ammonia excretion, and metal toxicity. *J of Exp Zool Part A: Ecol and Integr Physiol* **335,** 8-10 (2021).

7. Ulluwishewa, D., Anderson, R. C., McNabb, W. C., Moughan, P. J., Wells, J. M., & Roy, N. C. Regulation of tight junction permeability by intestinal bacteria and dietary components. *J Nutr* **141,** 769–776 (2011).
