# Supplementary material for: Important role of endogenous microbial symbionts of fish gills in the challenging but highly biodiverse Amazonian blackwaters
Source: Nat Commun. 2023 Jul 6;14:3903. doi: 10.1038/s41467-023-39461-x (PMC10326040; doi:10.1038/s41467-023-39461-x)
Supplement: Supplementary file 3 — Reporting Summary [file 41467_2023_39461_MOESM3_ESM.pdf]

nature portfolio

Corresponding author(s): François-Étienne Sylvain

Last updated by author(s): May 23, 2023

Reporting Summary

Nature Portfolio wishes to improve the reproducibility of the work that we publish. This form provides structure for consistency and transparency in reporting. For further information on Nature Portfolio policies, see our Editorial Policies and the Editorial Policy Checklist.

Statistics

For all statistical analyses, confirm that the following items are present in the figure legend, table legend, main text, or Methods section.

n/a	Confirmed	
☐	☒	The exact sample size (*n*) for each experimental group/condition, given as a discrete number and unit of measurement
☐	☒	A statement on whether measurements were taken from distinct samples or whether the same sample was measured repeatedly
☐	☒	The statistical test(s) used AND whether they are one- or two-sided<br>*Only common tests should be described solely by name; describe more complex techniques in the Methods section.*
☐	☒	A description of all covariates tested
☐	☒	A description of any assumptions or corrections, such as tests of normality and adjustment for multiple comparisons
☐	☒	A full description of the statistical parameters including central tendency (e.g. means) or other basic estimates (e.g. regression coefficient) AND variation (e.g. standard deviation) or associated estimates of uncertainty (e.g. confidence intervals)
☐	☒	For null hypothesis testing, the test statistic (e.g. *F*, *t*, *r*) with confidence intervals, effect sizes, degrees of freedom and *P* value noted<br>*Give P values as exact values whenever suitable.*
☒	☐	For Bayesian analysis, information on the choice of priors and Markov chain Monte Carlo settings
☐	☒	For hierarchical and complex designs, identification of the appropriate level for tests and full reporting of outcomes
☐	☒	Estimates of effect sizes (e.g. Cohen's *d*, Pearson's *r*), indicating how they were calculated

Our web collection on statistics for biologists contains articles on many of the points above.

Software and code

Policy information about availability of computer code

Data collection	No software was used.
Data analysis	Salmon v1.3.0, R v4.0.2 and SqueezeMeta v1.5.1 were used to analyze the data in our study. All the code used for data analysis and all inputs files are freely available on the Open Science Network platform (URL: https://osf.io/qea5j/).

For manuscripts utilizing custom algorithms or software that are central to the research but not yet described in published literature, software must be made available to editors and reviewers. We strongly encourage code deposition in a community repository (e.g. GitHub). See the Nature Portfolio guidelines for submitting code & software for further information.

Data

Policy information about availability of data

All manuscripts must include a data availability statement. This statement should provide the following information, where applicable:

- Accession codes, unique identifiers, or web links for publicly available datasets
- A description of any restrictions on data availability
- For clinical datasets or third party data, please ensure that the statement adheres to our policy

The datasets generated for the current study can be found in the Sequence Read Archive (SRA) repository, BioProjectIDs: PRJNA839167 [https://www.ncbi.nlm.nih.gov/bioproject/PRJNA839167/], PRJNA839174 [https://www.ncbi.nlm.nih.gov/bioproject/?term=PRJNA839174], PRJNA902364 [https://www.ncbi.nlm.nih.gov/bioproject/?term=PRJNA902364], PRJNA902723 [https://www.ncbi.nlm.nih.gov/bioproject/?term=PRJNA902723], PRJNA901905 [https://

www.ncbi.nlm.nih.gov/bioproject/?term=PRJNA901905], PRJNA902720 [https://www.ncbi.nlm.nih.gov/bioproject/?term=PRJNA902720], PRJNA902365 [https://www.ncbi.nlm.nih.gov/bioproject/?term=PRJNA902365], PRJNA902722 [https://www.ncbi.nlm.nih.gov/bioproject/?term=PRJNA902722], PRJNA902358 [https://www.ncbi.nlm.nih.gov/bioproject/?term=PRJNA902358] (data from gills), PRJNA736442 [https://www.ncbi.nlm.nih.gov/bioproject/?term=PRJNA736442] and PRJNA736450 [https://www.ncbi.nlm.nih.gov/bioproject/?term=PRJNA736450] (data from bacterioplankton). The NCBI "16S Microbial" database can be accessed here: https://www.ncbi.nlm.nih.gov/refseq/targetedloci/16S_process/.

Human research participants

Policy information about studies involving human research participants and Sex and Gender in Research.

Reporting on sex and gender	N/A
Population characteristics	N/A
Recruitment	N/A
Ethics oversight	N/A

Note that full information on the approval of the study protocol must also be provided in the manuscript.

Field-specific reporting

Please select the one below that is the best fit for your research. If you are not sure, read the appropriate sections before making your selection.

☐ Life sciences ☐ Behavioural & social sciences ☒ Ecological, evolutionary & environmental sciences

For a reference copy of the document with all sections, see nature.com/documents/nr-reporting-summary-flat.pdf

Ecological, evolutionary & environmental sciences study design

All studies must disclose on these points even when the disclosure is negative.

Study description

Field sampling: Four species of fish were collected from 15 sampling sites (20 specimens per species per site, totalizing 80 fish per site). There were 15 sampling sites which were distributed among 3 water types (5 blackwater sites, 7 whitewater sites and 3 clearwater sites). One gill sample was taken on every fish sampled (20 replicates per site). In some instances, it was not possible to collect all 20 specimens per species per site; the exact number of specimens per species per site is indicated in Supplementary Table 4. In this experiment, the "sampling site" factor was nested within the "water type" factor, since only one water type could be attributed to each sampling site. However, the "fish species" factor was crossed with the "water type" factor, since all the species were sampled in all water types.
Laboratory experiment: There were four experimental treatments of zebrafish, and each treatment was tested in two different water types (blackwater and whitewater). The "treatment" factor was crossed with the "water type" factor since all treatments were conducted in all water types. Each treatment had two replicate groups (total of 16 groups). Thus, the replicate groups were nested within the "treatment" factor. A total of 10 replicate fish were sampled from each group one time per experiment (=160 fish sampled per experiment), and the experiment was repeated two times (a total of 320 fish were sampled).

Research sample

Field sampling: Four species were sampled in the Brazilian Amazon: Flag cichlids (Mesonauta festivus), freshwater sardine (Triportheus albus), black piranha (Serrasalmus rhombeus) and peacock bass (Cichla spp.). These species were chosen because they are abundant in the Amazon and they are found in all the environments that define the hydrochemical gradient explored during this study. No information was collected on the sex or the age of the fish sampled.
Laboratory experiment: We collected zebrafish (Danio rerio) larvae raised in the laboratory. No information was collected on the sex of the fish sampled. All zebrafish samples were collected five days post-hatching.

Sampling strategy

One gill was sampled on every fish collected. We aimed to collect 20 biological replicates (20 individual fish) per species per site for the field experiment, and 10 replicates per group for the laboratory experiment. Previous studies on fish microbiomes have often only used between 3-5 fish replicates per site/ species/ treatment (see a review in Ghanbari et al. 2015, and a recent study Kim et al. 2021).

Ghanbari M, Kneifel W, Domig KJ. 2015. A new view of the fish gut microbiota: advances from next-generation sequencing. Aquaculture 448:464–475. doi: 10.1016/j.aquaculture.2015.06.033.

Kim PS, Shin NR, Lee JB, Kim MS, Whon TW, Hyun DW, Yun JH, Jung MJ, Kim JY, Bae JW. 2021. Host habitat is the major determinant of the gut microbiome of fish. Microbiome 9(1):166. doi: 10.1186/s40168-021-01113-x.

Data collection

Field sampling: Fish gill samples were collected by dissection directly on site, after fishing. One randomly chosen gill arch was cut using sterile scissors and sterile tweezers were used to place the samples in sterile 2 mL Eppendorf tubes containing NAP buffer. The the following authors: FÉS, AH, NL, DB and ND participated to sample collection. The downstream molecular analysis on these samples was conducted by: FÉS, NL, PLM, JC.

Laboratory experiment: Zebrafish gill samples were collected by dissection in the laboratory, in a sterile environment. In brief,

zebrafish heads were cut under a microscope using sterile scissors, and tweezers were then used to place the samples in sterile 2 mL Eppendorf tubes containing NAP buffer. The following authors conducted this sampling: FÉS and JC. Then, the molecular analysis on these samples was conducted by: FÉS, NL, PLM, JC.

Timing and spatial scale

To collect samples in the field, 15 expeditions were organized at the following sites:

Site #	Site name	GPS S	GPS W	Sampling time
1	Rio Negro - Barcelos	0°50'50.8"S	62°57'40.3"W	11/2018
2	Rio Negro - Santo Alberto	1°23'29.8"S	61°59'35.3"W	10/2019
3	Rio Negro - Anavilhanas	2°41'46.1"S	60°46'33.3"W	10/2018
4	Lago do cemeterio	3°02'16.6"S	60°32'42.7"W	10/2019
5	Lago Téfé	3°27'55.2"S	64°53'13.2"W	11/2019
6	Rio Branco	1°19'05.7"S	61°52'34.7"W	10/2019
7	Lago Janauari	3°12'03.4"S	60°03'10.1"W	10/2018
8	Lago Catalão	3°09'56.4"S	59°54'38.4"W	10/2018
9	Lago Janauaca	3°23'37.5"S	60°19'52.6"W	11/2018
10	Rio Manacapuru	3°16'16.9"S	60°42'03.2"W	11/2018
11	Lago Téfé-Solimões	3°21'07.4"S	64°40'21.4"W	11/2019
12	Lago des pirates	3°15'19.2"S	64°41'44.3"W	11/2019
13	Balbina Reservoir	1°50'55.9"S	59°34'59.5"W	10/2018
14	Rio Tapajós	2°18'57.8"S	55°00'45.0"W	10/2019
15	Rio Curua-Una	2°48'19.1"S	54°17'52.2"W	11/2018

These sites were chosen for their accessibility, their location (e.g. the distance from the city of Manaus (AM), Brazil), and their water type (5 blackwater sites, 3 clearwater sites and 7 whitewater sites).

Data exclusions

Field collection: If a fish was apparently sick or died before the collection of the samples, the fish was discarded.
Sequence analysis level: Contaminant 16S rRNA sequences were identified with the R package "decontam" with the default thresholds and were removed from the dataset.

Reproducibility

Field work: The fish collection was repeated 15 times, since 15 sampling sites were visited for our study. The same methodology was used to collect fish specimens (i.e. gill nets) at all sampling sites. There were also four fish species collected at every sampling site, thus the analysis on the effect of water type on fish microbiomes was replicated four times (once for each fish species). The results from Figure 2 suggest that all attempts at replication were successful: The microbiomes from the four fish species studied show a similar response to the "water type" factor (i.e. the same bacterial clades are overly abundant in blackwater specimens, for the four fish species collected).

Laboratory experiment: The laboratory experiment (raising the fry and collecting the gills of zebrafish larvae) was repeated successfully two times. All attempts at replication were successful: The same zebrafish larvae mortality rates were observed for each treatment between the replications of the experiment.

Randomization

Field work: The first 20 fish of each desired species that were caught in the gill nets were sampled, irrespectively of their size and sex.
Laboratory experiment: All fish eggs came in the same vial, all eggs were sterilized together and larvae were all raised in the same sterile vial. After four days, the larvae were randomly distributed in the vials that contained the water specific to each treatment. All larvae had the same size and it was impossible to determine sex at that stage.
Before sequencing, all RNA/DNA extracts from the field and the laboratory were randomly distributed on the plates sent for sequencing, so that plate-specific bias would not interfere with the species-specific, site-specific or treatment-specific signals studied here.

Blinding

Blinding was not very relevant to this study, as we worked with randomly collected/distributed fish samples. Nevertheless, some blinding was done after RNA/DNA extractions of the gill samples: The staff in charge of the molecular analyses were unaware of the fish host species, localization, and/or group of origin (e.g. sterile or non-sterile group in the zebrafish experiment) of the samples they were working with, as all samples were labeled with PCR-plate-specific IDs (e.g. A1, A2, A3, etc.). This ensured that samples from different origins (and even extraction blanks) were processed in the same manner by the staff doing the molecular analyses.

Did the study involve field work? ☒ Yes ☐ No

Field work, collection and transport

Field conditions

Field work was always carried during the dry season in the Amazon (September to December) in 2018 and 2019. This season is characterized by elevated temperatures (approximately 30°C) and low rainfall. The exact timing and location of the sampling sites is detailed in the above section "Timing and spatial scale". Weather conditions were homogeneous between the different sampling sites.

Location

The exact location of the sampling sites is detailed in the above section "Timing and spatial scale". A total of 34 hydrochemical parameters (e.g. pH, conductivity, dissolved oxygen, dissolved organic carbon, various metals, etc.) were measured at each sampling site. These values are detailed in Tables S5-8 in Supplementary Material (too much data to include in this form).

Access & import/export

All samples were collected as authorized by the Ethics Committee for the Use of Animals of the Brazilian National Institute of

Access & import/export	Research of the Amazon (Manaus, Brazil) (permit # 29837-14, issue date 15/12/2017) and the Animal Protection Committee of Laval University (Quebec, Canada) (permit # 2018021-1, issue date 06/29/2018).
Disturbance	The disturbance caused by our study was minimal, since only 20 specimens for every targeted species were collected per site. When additional specimens or species were collected, they were immediately discarded and released alive. After setting up the gill nets, they were checked every 15 minutes to minimize the by-catch and the suffering of fish that were caught. Gill nets were set up in strategic locations to target the species of interest for this study, and minimize the disturbance to other species found in the same habitat.

Reporting for specific materials, systems and methods

We require information from authors about some types of materials, experimental systems and methods used in many studies. Here, indicate whether each material, system or method listed is relevant to your study. If you are not sure if a list item applies to your research, read the appropriate section before selecting a response.

Materials & experimental systems

n/a	Involved in the study
☒	☐ Antibodies
☒	☐ Eukaryotic cell lines
☒	☐ Palaeontology and archaeology
☐	☒ Animals and other organisms
☒	☐ Clinical data
☒	☐ Dual use research of concern

Methods

n/a	Involved in the study
☒	☐ ChIP-seq
☒	☐ Flow cytometry
☒	☐ MRI-based neuroimaging

Animals and other research organisms

Policy information about studies involving animals; ARRIVE guidelines recommended for reporting animal research, and Sex and Gender in Research

Laboratory animals	We raised sterile larvae of wild-type zebrafish (Danio rerio) for the laboratory experiment. The age of the animals sampled was 5 days old.
Wild animals	Four species were sampled in the Brazilian Amazon: Flag cichlids (Mesonauta festivus), freshwater sardine (Triportheus albus), black piranha (Serrasalmus rhombeus) and peacock bass (Cichla spp.). Fish were collected using gill nets and all by-catch was immediately discarded and released alive back in the environment. After collection, live specimens were put in coolers with environmental water (approx. 100L) and two air pump systems (for oxygenation) to ensure fish survival during travel from the sampling site to the field laboratory (approx. 30 minutes). After arrival at the field laboratory, fish were immediately euthanized and dissected to collect the gills. The ages of the animals sampled are unknown.
Reporting on sex	Fish were randomly sampled (in the field and in the laboratory experiments), without regards to sex. Sex was not determined (and often could not be determined due to size or anatomical considerations), thus no sex-specific analyses were conducted in this project.
Field-collected samples	After dissection of the fish, gill samples were stored in 2 mL of NAP buffer. This nucleic acid preservation buffer preserves the integrity of DNA and RNA in biological tissue samples up to several months at room temperature (see Camacho-Sanchez et al. 2013). The 2 mL vials of NAP buffer containing the samples were then stored at -20°C and transported on ice from the field laboratory to the main laboratory where molecular analyses were conducted. After DNA/RNA extractions, the extracts were stored at -80°C.<br><br>Camacho-Sanchez, M., Burraco, P., Gomez-Mestre, I., & Leonard, J. A. Preservation of RNA and DNA from mammal samples under field conditions. Mol Ecol Res 13, 663-673 (2013).
Ethics oversight	All samples were collected as authorized by the Ethics Committee for the Use of Animals of the Brazilian National Institute of Research of the Amazon (Manaus, Brazil) (permit # 29837-18) and the Animal Protection Committee of Laval University (Quebec, Canada) (permit # 2018021-1).

Note that full information on the approval of the study protocol must also be provided in the manuscript.
